# Past and Current Perspectives in Modeling Bacteria and Blood–Brain Barrier Interactions

**DOI:** 10.3389/fmicb.2019.01336

**Published:** 2019-06-13

**Authors:** Brandon J. Kim, Eric V. Shusta, Kelly S. Doran

**Affiliations:** ^1^Institute for Hygiene and Microbiology, University of Würzburg, Würzburg, Germany; ^2^Department of Chemical and Biological Engineering, University of Wisconsin, Madison, WI, United States; ^3^Department of Immunology and Microbiology, University of Colorado School of Medicine, Aurora, CO, United States

**Keywords:** bacteria, blood–brain barrier, meningitis, stem cells, brain endothelial cell

## Abstract

The central nervous system (CNS) barriers are highly specialized cellular barriers that promote brain homeostasis while restricting pathogen and toxin entry. The primary cellular constituent regulating pathogen entry in most of these brain barriers is the brain endothelial cell (BEC) that exhibits properties that allow for tight regulation of CNS entry. Bacterial meningoencephalitis is a serious infection of the CNS and occurs when bacteria can cross specialized brain barriers and cause inflammation. Models have been developed to understand the bacterial – BEC interaction that lead to pathogen crossing into the CNS, however, these have been met with challenges due to these highly specialized BEC phenotypes. This perspective provides a brief overview and outlook of the *in vivo* and *in vitro* models currently being used to study bacterial brain penetration, and opinion on improved models for the future.

## The Brain Barriers

The brain barriers are comprised of a number of endothelial and epithelial cellular barriers that serve to maintain brain homeostasis and prevent pathogen entry into the central nervous system (CNS; Engelhardt and Sorokin, 2009; Abbott et al., 2010; Redzic, 2011). These barriers are especially adept at regulating nutrients and molecules passage into the CNS, while at the same time restricting access to many xenobiotic drugs and pathogens (Engelhardt and Sorokin, 2009; Abbott et al., 2010; Redzic, 2011; Spindler and Hsu, 2012; Doran et al., 2016). In the average adult human, the total cerebral microvasculature has been estimated at a total surface area of 15–25 m^2^, and a total length of around 600 km (Zlokovic, 2005; Wong et al., 2013). The endothelial blood–brain barrier (BBB) makes up the majority of the brain microvasculature and is the primary location for exchange processes between the circulation and CNS (Redzic, 2011). The BBB is supported by a number of other cell types, such as astrocytes, pericytes, and neurons [collectively called the neurovascular unit (NVU)], that not only enhance barrier properties, but also regulate the ability of the endothelial cell to transport essential nutrients into the CNS and discharge metabolic products from the CNS into the blood (Abbott et al., 2010). The endothelium of the BBB possesses barrier properties different than other vascular networks elsewhere in the body, and these phenotypes contribute to their ability to function. The meningeal blood-cerebral spinal fluid barrier (mBCSFB) is an additional endothelial barrier comprised of brain vessels with similar properties to the BBB, although not supported by the NVU and associated cell types. Instead, the endothelium of the mBCSFB is surrounded by cerebral spinal fluid (CSF), and can be closely associated with leptomeningeal cells (Rua and McGavern, 2018). An important epithelial brain barrier can be found in the choroid plexus (CP) where the capillaries are more similar to those of the peripheral endothelium exhibiting fenestrations and do not form a barrier, instead a single layer of specialized epithelial cells forms the barrier phenotype of the CP. These epithelial cells are the principle component of what is known as the blood-CSF barrier (BCSFB; Engelhardt and Sorokin, 2009). One of the major phenotypes that describe each of these brain barriers is the presence of tight junctions that seal adjacent cells to one another, and represent a distinctive barrier phenotype of the brain barriers (Engelhardt and Sorokin, 2009). The tight junctions are formed by a host of transmembrane proteins including occludin, ZO-1 and claudin-5 (Nitta et al., 2003). To cause serious CNS infection, infectious agents such as bacteria, viruses, and parasites, must cross or subvert these brain barriers.

Bacterial meningoencephalitis is a serious infection of the CNS and is uniformly fatal if left untreated. Certain bacteria that cause meningoencephalitis have the ability to interact with and penetrate one or more of the brain barriers allowing access to the CNS and causing inflammation. This perspective outlines models utilized to examine bacterial pathogen–brain barrier studies, and discusses the incorporation of novel barrier models with particular focus on the endothelial barriers of the CNS and the key bacterial pathogens associated with meningoencephalitis and CNS disease.

## Bacterial Pathogens Associated With Cns Disease

There are a number of bacterial pathogens capable of interacting with and penetrating brain endothelial cells (BECs) to gain access to the CNS and cause meningoencephalitis. *Neisseria meningitidis* (meningococcus) and *Streptococcus pneumoniae* (pneumococcus) are leading causes of meningoencephalitis in young adults and the elderly (Doran et al., 2016; Coureuil et al., 2017). *N. meningitidis* is a leading cause of epidemic meningococcal meningoencephalitis in the “meningitis belt” of sub-Saharan Africa and developing nations, but remains a health threat in the developed world (Doran et al., 2016; Schubert-Unkmeir, 2017). *Streptococcus pneumonaie* (pneumococcus) remains a common cause of bacterial meningoencephalitis despite the development of multivalent vaccines (Feikin et al., 2013; Doran et al., 2016). Meningeal pathogens that affect the newborn include *Streptococcus agalactiae*, commonly known as Group B *Streptococcus* (GBS), and *Escherichia coli* K1. GBS is the leading cause of neonatal meningoencephalitis and can be vertically transmitted from mother to newborn during the birthing process (Doran and Nizet, 2004). While prophylactic antibiotic treatment has limited maternal GBS colonization and transmission to the newborn, treatment has had little impact on the incidence of GBS meningoencephalitis (Berardi et al., 2013). *E. coli* K1 is the second leading cause of neonatal meningoencephalitis. While modern antibiotic treatments have reduced mortality, complications remain due to the amount of endotoxin that remains from dead bacteria resulting in septic shock (Shenep and Mogan, 1984; Doran et al., 2016). Some additional bacterial pathogens may under specific conditions or in certain hosts cause CNS disease, such as *Listeria monocytogenes*, *Mycobacterium tuberculosis*, *Streptococcus suis*, *Salmonella* spp., *Klebsiella* spp., and *Staphylococcus aureus*. In general, many of these pathogens can persist in the human as a normal colonizer of mucosal surfaces. However, in certain cases can enter the blood stream, replicate to high levels, and interact with brain barriers where they may gain access to the CNS (Doran et al., 2016). Presently various models to understand how these pathogens enter the CNS have been employed and better appropriations of cellular brain barriers are being developed.

## *In Vivo* Models Used to Study Brain Penetration and Cns Disease

To elucidate mechanisms of penetration of the BBB by pathogens in the context of live organisms, researchers utilize *in vivo* models of CNS transit and pathology. These models have the advantage of possessing intact brain barriers, and are conducted in the context of a competent immune system. Much work has been done to elucidate mechanisms of BBB dysfunction during infection using mouse, rat, rabbit, and zebrafish models, and each model has distinct advantages and disadvantages. Identifying *in vivo* mechanisms of brain barrier traversal is a critical step in understanding the pathogenesis of many meningeal pathogens.

Rodent models have been widely utilized to study bacterial meningoencephalitis, specifically the mouse and rat. Typically, these models can be distinguished mainly by the route of injection. Direct infection of the CNS through intra-cerebral or intra-cisternal route allows for the monitoring of host–pathogen interactions once the pathogen is in the CNS as well as leukocyte transmigration (Shapiro et al., 2000; Koedel et al., 2001; Grandgirard et al., 2007; Reiß et al., 2011; Koopmans et al., 2018). While this method is useful, it lacks a more natural course of dissemination through the blood stream (Chiavolini et al., 2008). To this end, hematogenous routes have been employed introducing bacteria via intraperitoneal, intravenous, or intracardiac injections. This route allows for the development of bacteriemia prior to crossing of the brain barriers and better models a blood to CNS transit of bacteria (Ferrieri et al., 1980; Kim et al., 1992, 2015a; Tan et al., 1995; Hoffman et al., 2000; Wang and Kim, 2002; Liu et al., 2004; Doran et al., 2005; Banerjee et al., 2010). Alternate routes of infection leading to meningoencephalitis have been utilized such as intranasal administration of bacteria (Ferrieri et al., 1980; Zwijnenburg et al., 2001; Mittal et al., 2010, 2011). This method can be especially useful when examining a pathogen that typically colonizes the nasopharynx and surrounding tissue such as with *S. pneumoniae* (Zwijnenburg et al., 2001). In the case of food-borne pathogens, such as *L. monocytogenes*, intragastric infection can be useful to mimic disease (Lecuit et al., 2001; Maury et al., 2016). Recently, an interesting mouse model for GBS infection was employed that relied on a colonized mother passing along GBS to neonates vertically as it is hypothesized to take place in human infection (Andrade et al., 2018). While extremely useful and widely used, rodent models have their limitations especially when it comes to pathogens such as *L. monocytogenes* and *N. meningitidis* which are primarily human specific pathogens. In order to model disease with these pathogens, transgenic mice which express specific human genes or humanized mice are often utilized which limits the scope of the interactions that may be studied (Mackinnon et al., 1992; Lecuit et al., 2001; Yi et al., 2003; Zarantonelli et al., 2007; Johswich et al., 2013; D’Orazio, 2014; Maury et al., 2016).

In addition to rodent models, other *in vivo* systems have been developed to study bacterial penetration of brain barriers and progression of meningoencephalitis. The zebrafish (*Danio rerio*) has certain advantages such as high group numbers, optically clear, and genetically tractable. In recent years zebrafish have been used to model brain barrier crossing or meningoencephalitis for *Mycobacterium*, GBS, *S. pneumoniae*, and *Streptococcus iniae* (Patterson et al., 2012; Harvie et al., 2013; van Leeuwen et al., 2014; Kim et al., 2015a,b; Jim et al., 2016). Interestingly pathogens like *S. iniae* and GBS are zoonotic pathogens impacting fish in aquaculture with pathology similar to humans, as it can cause meningoencephalitis in fish (Delannoy et al., 2013; Wang et al., 2017; Mishra et al., 2018). Thus, the zebrafish model presents an interesting method to examine bacterial pathogenesis in fish as well to improve aquaculture methods. However, it is important to note that the zebrafish BBB differs from the mammalian BBB particularly due to the lack of true astrocytes, instead they possess a different glial cell type that serves a similar purpose (O’Brown et al., 2018). Other less common mammalian models include the rabbit, guinea pig, and the gerbil to model meningoencephalitis in a wide range of pathogens (Michael Scheld et al., 1979; Sinai et al., 1980; Sulc et al., 1992; Zysk et al., 1996; Blanot et al., 1997; Tauber, 1998; Marra and Brigham, 2001; Chiavolini et al., 2008; D’Orazio, 2014; Tucker et al., 2016). In certain cases, non-human primates have been used and demonstrate natural meningoencephalitis, especially with *L. monocytogenes* (Kaufmann and Quist, 1969; Mook-kanamori et al., 2011; Lemoy et al., 2012; Scanga and Flynn, 2014). However, these models are often cost and facilities-prohibitive and difficult to get approved due to institutional restrictions. Each *in vivo* model offers specific advantages and/or disadvantages and selection of models may come down to availability and usefulness to the research question.

## The Use of *In Vitro* Models of Endothelial Brain Barriers During Infection

*In vitro* models comprised of human BECs eliminate some of the pitfalls from the *in vivo* models such as interspecies differences, scalability, and availability (Syvänen et al., 2009; Warren et al., 2009; Lippmann et al., 2012). One of the first immortalized cell lines utilized for study of bacterial BEC interaction was the human brain microvascular endothelial cell (hBMEC) line. Cells were initially isolated from a patient with epilepsy and then immortalized by the transformation with SV40 large T-antigen (Nizet et al., 1997). The hBMEC line has been used widely for the determination of bacterial-BEC interactions with bacterial pathogens such as *S. pneumoniae*, GBS, *N. meningitidis*, *E. coli* K1, *L. monocytogenes*, and others (Nizet et al., 1997; Greiffenberg et al., 1998; Badger et al., 1999, 2002; Hoffman et al., 2000; Stins et al., 2001; Doran et al., 2005; Uchiyama et al., 2009; Banerjee et al., 2010; Miraglia et al., 2018). While this model has been used successfully over the years for mechanistic studies, it has some limitations including the lack of certain BEC key characteristics such as continuous tight junction staining and claudin-5 expression (Table 1; Kim et al., 2017). Other immortalized BECs such as hCMEC/D3 have been developed by transforming microvessels isolated from an epileptic patient, with hTERT and SV40 (Weksler et al., 2005). This model has been employed to examine interactions with various infectious agents ranging from bacteria, viruses, fungi, and parasites (Coureuil et al., 2010; Bernard et al., 2014; N’Dilimabaka et al., 2014; Volle et al., 2015; Wang et al., 2015; Mutso et al., 2017; Aaron et al., 2018; Deng et al., 2018; Jiménez-Munguía et al., 2018). The hCMEC/D3 model better recapitulates more phenotypes of BECs, such as claudin-5 expression, however, they still do not localize certain tight junction components and produce relatively low transendothelial electrical resistance (TEER), indicative of suboptimal barrier function (Weksler, 2005; Eigenmann et al., 2013). Each *in vitro* model using primary or immortalized BECs has differing attributes that must be carefully assessed depending on envisioned application, and a comprehensive review can be found elsewhere (Helms et al., 2015).

**Table 1:** Comparison of *in vitro* BEC models.

Model	Type	TEER (Ω × cm^2^)	Tight junctions	Bacterial pathogens studied	Model origin (year)	References
hBMEC	Human immortalized by SV40 large T antigen	20 to 40	ZO-1 and Occludin present. Claudin-5 absent	*S. pneumoniae*, *S. agalactiae*, *E. coli Kl*, *L. monocytogenes*, *N. meningitidis*, *B. abortus*, *C. freundii*, and others	1997	Badger et al., 1999; Banerjee et al., 2010; Doran et al., 2005; Eigenmann et al., 2013; Greiffenberg et al., 1998; Helms et al., 2015; Hoffman et al., 2000; Badger et al., 2002; Miraglia et al., 2018; Nizet et al., 1997; Stins et al., 2001; Uchiyama et al., 2009
hCMEC/D3	Human immortalized by SV40 large T antigen and hTERT	10 to 200	ZO-1, Occludin, and Claudin-5 present	*N. meningitidis*, *S. agalactiae*, *S. pneumoniae*, *S. suis*	2005	Bernard et al., 2014; Coureuil et al., 2010; Deng et al., 2018; Eigenmann et al., 2013; Helms et al., 2015; Jiménez-Munguía et al., 2018; Wang et al., 2015; Weksler et al., 2005
iPSC-derived BECs	Human stem-cell derived	250 to 5000+	ZO-1, Occludin, and Claudin-5 present and localized to cell-cell junctions	*S. agalactiae*, *N. meningitidis*	2012	Gomes et al., 2019; Helms et al., 2015; Hollmann et al., 2017; Kim et al., 2017; Lippmann et al., 2012, 2014; Qian et al., 2017; Stebbins et al., 2016

## Improved *In Vitro* Models and Future Outlooks

**FIGURE 1:**
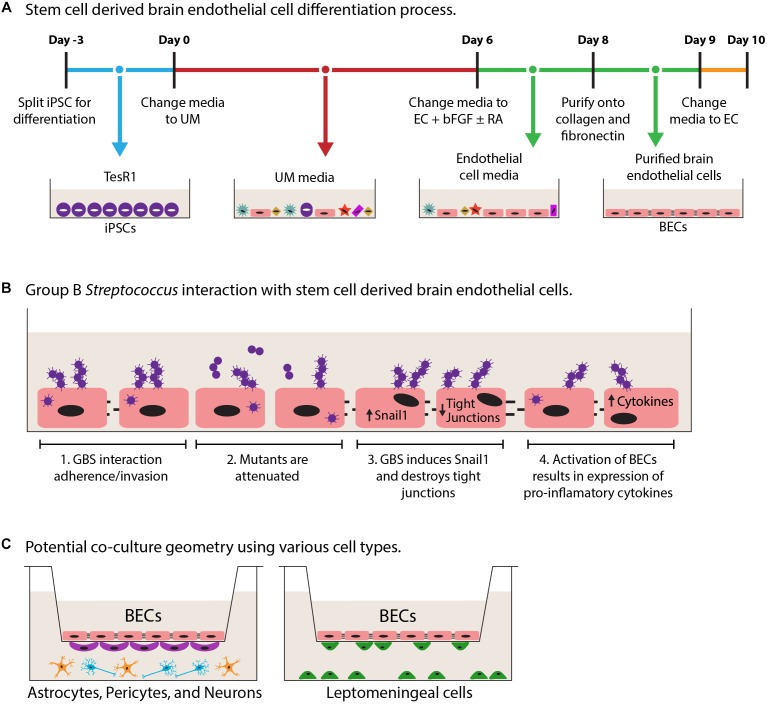
Schematic diagrams of iPSC-derived BEC models and co-culture. **(A)** Derivation of iPSC-derived BECs adapted from Lippmann et al. (2012). Overall the 13-day process results in purified iPSC-derived BECs. Day-3 to 0 iPSCs are expanded in TesR1 media. Day 0–6, cells are differentiated in unconditioned media (UM). Days 6–9, endothelial cells are expanded using endothelial cell (EC) media supplemented with basic fibroblast growth factor (bFGF), and retinoic acid (RA). On day 8 endothelial cells are purified onto a collagen/fibronectin matrix. Day 9 cells get a final change into EC media. **(B)** Schematic GBS infection of iPSC-BECs as described in Kim et al. (2017). B1, demonstrated that GBS can interact with iPSC-BECs by adherence, invasion, and intracellular survival. B2, GBS mutants are attenuated for iPSC-BEC interaction. B3, GBS induces the tight junction repressor Snail1 previously shown to contribute to the destruction of tight junctions. B4, innate immune activation is seen through the upregulation of cytokines and chemokines. **(C)** Potential transwell model combinations in co-culture with iPSC-derived BECs with other cell types of the CNS such as astrocytes (orange), neurons (blue), pericytes (purple), and leptomeningeal cells (green).

As mentioned above, many of the primary and immortalized BBB models lack critical BEC phenotypes limiting their usefulness (Helms et al., 2015). This highlights the challenge of modeling human BECs *in vitro*, as many of the models may lack key BBB properties. Primary cells may have low availability and once removed from the brain microenvironment, lose phenotypes associated with BECs (Lippmann et al., 2012; Helms et al., 2015). Additionally while immortalized BECs are highly scalable, they only offer a facile model and often lack continuous tight junctions and appropriate barrier function (Table 1; Lippmann et al., 2013; Helms et al., 2015; Rahman et al., 2016). In our opinion, recent advances in stem cell-based technologies offer the prospects of superior BBB modeling. These human stem cell-derived BECs possess expected markers, functional transporters, and continuous tight junction staining corresponding to the high TEER values (Table 1; Lippmann et al., 2012, 2014; Cecchelli et al., 2014; Stebbins et al., 2016; Hollmann et al., 2017; Qian et al., 2017). Using human induced pluripotent stem cells (iPSCs), BECs can be generated without the requirement of co-culture with other NVU cell types, and iPSC sources offer the added benefit of being easily scalable (Lippmann et al., 2012, 2014). Figure 1A provides an example of one differentiation process to achieve BECs from iPSC sources that has been utilized (Lippmann et al., 2012, 2014). The iPSC model has been used to examine drug delivery, genetic human disease and ischemic stroke (Clark et al., 2016; Vatine et al., 2016; Kokubu et al., 2017; Lim et al., 2017), but only recently has it been evaluated for the study of microbe interactions. It was shown that GBS adhere to, invade, and activate the iPSC-derived BECs, while GBS mutants known to have diminished BBB interaction were attenuated in the iPSC-derived model (Figure 1B; Kim et al., 2017). GBS infection also resulted in the disruption of tight junction components, ZO-1, Occludin, and Claudin-5 (Figure 1B). Furthermore, it was shown that a mechanism of GBS destruction of tight junctions through the Snail1 transcription factor was consistent in the iPSC-BEC model (Kim et al., 2017). While this mechanism was first shown using GBS, recently another meningeal pathogen, *E. coli* K1, has been found to induce Snail1 (Kim et al., 2015a; Yang et al., 2016). We anticipate that this mechanism of tight junction disruption may be utilized by other pathogens, and the iPSC-BEC model, with superior tight junction and barrier function would be an ideal system for conducting these studies. This demonstrates that the iPSC-derived BBB model can be utilized to study BBB interaction with bacterial CNS pathogens, and has the potential to be expanded to other pathogens. Interestingly, researchers have used iPSC-derived BECs to demonstrate that Zika virus can cross BECs without disrupting the junctions (Alimonti et al., 2018). A recent proof of principle publication showed that iPSC-BECs can also model interactions with the human specific *N. meningitidis* (Gomes et al., 2019). Thus, human iPSC-derived BEC models hold promise for further study of human specific pathogens, particularly those that lack robust *in vivo* models.

Advances in biological engineering have also paved the way for the modeling of other aspects of the brain barriers such as shear stress to mimic blood flow, and co-culture with supporting cell types of the NVU (e.g., astrocytes, pericytes, and leptomeningeal cells). BECs respond to cues from NVU by enhancing BBB phenotypes such as TEER, and tight junctions. Co-culture models have utilized the Transwell system and demonstrated enhanced properties with stem cell derived ECs and BECs. (Lippmann et al., 2012; Cecchelli et al., 2014; Appelt-Menzel et al., 2017; Canfield et al., 2017). It would thus be possible to model bacterial interaction during co-culture with enhanced BBB properties and potential cross talk between cells (Figure 1C). Direct interactions with glial cell types such as astrocytes and microglia have been explored, and in general it appears that glial cells respond by secreting cytokines and chemokines (Cooley et al., 2014; Stoner et al., 2015; Seele et al., 2016). Additionally, interactions at the mBCSFB would occur between bacteria and BECs that are supported by leptomeningeal cells, nevertheless until now only direct interactions with a cancerous leptomeningeal cell line have been investigated (Fowler et al., 2004; Alkuwaity et al., 2012). While responses of these other brain residing cell types have been established, little has been done to determine the impact of the other brain cell types in co-culture with BECs during infection (Figure 1C). Presently, work is being conducted to model the 3-dimentional blood vessel, and presents an opportunity for the incorporation of the natural vessel architecture (Adriani et al., 2015; Cho et al., 2015). These models take advantage of using flow and multicellularity while retaining the overall structure of a blood vessel. It will be of great interest to the field to understand the contribution of these other CNS cell types and architecture, to host–pathogen interaction and overall maintenance of barrier integrity during infection. Establishing multi-cellular *in vitro* systems that include high barrier forming endothelial cells, such as the iPSC-BECs, will expand our understanding of the host–pathogen interface at brain barriers and CNS infectious disease.

## Conclusion

Challenges in modeling host–pathogen interaction at the brain barriers have led to a variety of models both *in vitro* and *in vivo*. While *in vivo* models inherently have interspecies variations, widely used *in vitro* models of BECs generally lack robust BBB-like properties. We believe that an iPSC-derived BEC model may prove useful especially when high barrier function is required, or when a human-specific pathogen is used that lacks good *in vivo* models.

## Author Contributions

All authors listed have made a substantial, direct and intellectual contribution to the work, and approved it for publication.

## Conflict of Interest Statement

The authors declare that the research was conducted in the absence of any commercial or financial relationships that could be construed as a potential conflict of interest.
